# 原发性气管肿瘤的诊断与治疗进展

**DOI:** 10.3779/j.issn.1009-3419.2011.01.12

**Published:** 2011-01-20

**Authors:** 军 陈

**Affiliations:** 300052 天津，天津医科大学总医院肺部肿瘤外科；天津市肺癌研究所，天津市肺癌转移与肿瘤微环境重点实验室 Department of Lung Cancer Surgery, Tianjin Key Laboratory of Lung Cancer Metastasis and Tumor Microenvironment, Tianjin Lung Cancer Institute, Tianjin Medical University General Hospital, Tianjin 300052, China

原发性气管肿瘤是指起源于环状软骨至隆突平面的气管肿瘤，临床上非常少见，目前相关文献多为个案报道、单中心的病例回顾以及流行病学现况调查。美国癌症研究所（National Cancer Institute, NCI）的SEER数据库中，1973年-2004年共有574例原发性气管肿瘤，发病率约为每年新发病例为2.6/10万人^[1]^。原发性气管肿瘤约占所有恶性肿瘤的1%-3.5% ^[2]^，其发病率在呼吸系统肿瘤中约占0.2%，男女之比约为4:1，多见于成人，儿童原发性气管肿瘤以良性居多，良性率可达90% ^[3]^。与胸部的其它肿瘤，如肺癌、喉癌及食管癌的气管周围淋巴结转移和纵隔淋巴瘤侵犯气管相比，原发性气管肿瘤的发病率只有这些转移性病变的0.1%^[4]^。

## 1 原发性气管肿瘤的临床特征

原发性气管肿瘤的早期症状不明显，缺乏特异性的症状和体征，表现为刺激性咳嗽、咳痰、喘息、活动后胸闷、气短、痰中带血等非特异性症状，常常被误诊为肺部感染、支气管哮喘等，误诊率达72%，其中31%误诊半年以上^[5]^。

一般当瘤体占气管内径的2/3-3/4时，便会造成严重的呼吸道梗阻，直接威胁患者生命。原发性气管肿瘤的临床表现有以下特点^[6]^：①具有呼吸道梗阻的症状和体征，如吸气末和呼气初的哮鸣；呼吸困难，随体位变动加重或减轻；肿瘤严重阻塞气道时，出现口唇粘膜、甲床发绀和其它缺氧症状体征；②声嘶，肿瘤侵犯喉返神经或发生淋巴结转移压迫神经所致，声带麻痹加重呼吸困难；③咯血，早期多表现为痰中带少量血丝；④反复发生肺炎。

## 2 原发性气管肿瘤的病理类型

气管肿瘤来源于上皮细胞的有鳞癌，乳头瘤，来自上皮粘膜腺体的有腺样囊性癌；来自Kultschitzky细胞的有类癌，来自中胚组织的有平滑肌瘤、软骨瘤、血管瘤、错构瘤、神经纤维瘤等，来自几个胚层组织的有畸胎瘤。

气管肿瘤按恶性程度可分为恶性、低度恶性及良性三种。恶性的有鳞癌、腺癌及分化不良型癌，以鳞癌最多见；低度恶性肿瘤有腺样囊性癌、粘液类上皮癌及类癌，以腺样囊性癌多见；良性气管肿瘤有平滑肌瘤、错构瘤、乳头瘤、神经纤维瘤、涎腺混合瘤、血管瘤等。气管良性肿瘤的比例不到10%^[7]^。

Gaissert等^[8]^报道了美国麻省总医院40多年收治的357例气管肿瘤患者，共有360个肿瘤（3例患者存在两种不同类型的肿瘤），其中恶性肿瘤326个（90.6%），鳞癌135个（37.5%），腺样囊性癌135个（37.5%），类癌11个（3.1%），肉瘤14个（3.9%），良性肿瘤34个（9.4%）。徐松涛等^[9]^报道了复旦大学中山医院胸外科24年手术治疗的129例原发性气管肿瘤，术后病理结果为恶性肿瘤117例（90.7%），包括腺样囊性癌63例（48.8%），鳞癌45例（34.9%），良性肿瘤12例（9.3%）。

## 3 原发性气管肿瘤的临床评估和分期

原发性气管肿瘤的临床评估包括局部肿瘤的评估、明确病理及分期。影像学检查可以确定局部肿瘤的精确位置、范围和浸润程度。由于气管前后有纵隔组织及骨骼影的重叠，气管肿瘤在普通胸片上多为阴性。直接数字化X线摄影术（direct digital radiography, DDR）或高千伏正侧位片是显示腔内肿瘤有效而又简便的方法。这种方法所获图像较透亮、层次感强，但欠清晰。CT由于密度分辨率高，能较清楚地显示肿瘤向腔外侵犯纵隔组织、纵隔淋巴结转移情况等。随着螺旋CT的产生及图像后处理软件的成熟，例如通过CT仿真内镜（CT virtual endoscopy, CTVE）、表面遮盖显示（shaded surface display, SSD）、MPR等技术处理，所获图像能多方位显示气管肿瘤。MRI摄影形成的冠状面、矢状面、横断面三维图像对显示气管肿瘤及肿瘤与周围组织的关系比其它检查方法更清楚、更全面^[10]^。

纤维支气管镜可以更为准确直接地评估肿瘤的长度和未受累的气管，而且可以进行活组织检查和治疗。气管超声内镜可以提供更多气管壁厚和气管外肿瘤的侵犯情况的信息。但对于气管严重梗阻的患者，内镜操作可能影响通气，甚至引起窒息死亡的危险^[11]^。

同其它实体肿瘤一样，远处转移的评估手段包括头部核磁共振、躯干部位的CT检查以及PET检查。PET的价值取决于肿瘤的类型和分级，鳞癌对示踪剂有不均一的高摄取，而腺样囊性癌和粘液表皮样癌的摄取则依赖于肿瘤的分化程度^[12]^。据Gaissert^[13]^统计，对选择手术的病例，约10%的患者发现有远处转移，而对于非选择的患者，其比例更高，因此对于原发性气管肿瘤的全身情况评估非常重要。

目前，气管原发性恶性肿瘤的病理分期尚无明确定义。Webb等^[14]^通过回顾分析74例气管原发性肿瘤病例，提出了一个简单实用的分型建议。T1：肿瘤直径 < 2 cm，局限于气管内；T2：肿瘤直径 > 2 cm，局限于气管内；T3：起源于气管但侵犯至气管外，但无其它器官受累；T4：肿瘤侵犯周围器官。此外有淋巴结转移为N1，没有为N0。有远处转移为M1，否则为M0。

## 4 原发性气管肿瘤的外科治疗

原发性气管肿瘤进展比较缓慢，大多数病例仅在其病程晚期才发生转移，因此对没有转移的气管肿瘤或/和需要解除气道梗阻的患者都应争取外科手术切除治疗。

气管肿瘤的手术方法包括肿瘤局部切除、窗形切除、袖状切除端-端吻合术等。气管良性肿瘤可考虑肿瘤局部切除、窗形切除等相对保守的术式。而袖状切除端-端吻合术是目前大家普遍接受的、最理想的完整切除气管肿瘤的手术方式^[15]^。而在手术入路方面，一般认为上段气管肿瘤多采用颈部衣领状横切口，必要时可加上段胸骨纵行切开入口；中段气管肿瘤多采用胸骨正中切口；下段气管肿瘤和大多数隆凸肿瘤，均采用右胸后外侧切口^[16]^。

对于侵犯隆突的气管肿瘤，需行隆突重建术。隆突重建术主要有三种方式：将左右主支气管缝合成新的隆突，然后再将气管与此新隆突缝合重建；或将气管与一侧主支气管端端吻合，再将另侧主支气管与气管侧壁行端侧吻合；也可以将气管与一侧主支气管行端端吻合后，再将另侧主支气管与吻合后的主支气管行端侧吻合^[7]^。

关于气管的安全切除长度问题目前尚无定论，需要考虑患者的年龄、颈部的活动度、体重等。颜昱等^[17]^报道可直接吻合的气管切除最大长度为6.8 cm。手术气管切除长度越长，吻合张力越大，术后吻合口并发症发生率越高。一般认为切除的安全长度为4.0 cm，对端吻合基本无困难，超过4.0 cm者需用各种气管游离和松解的方法以减少吻合口张力，避免造成吻合张力过大导致吻合口裂开。

Grillo等^[18]^通过尸检研究发现，安全切除的气管长度为6.4 cm，约13.4个气管环，占气管长度的56.7%，随着切除长度的增加，吻合口张力呈指数曲线增加。其中，报道了主要的下部气管游离松解方式：游离右肺门，松解下肺韧带，可以增加3 cm（2.0 cm-4.2 cm）气管切除长度；切断左主支气管，行左主支气管-右中间支气管端侧吻合重建，可以增加2.7 cm（1.5 cm-6.5 cm）气管切除长度；切开心包，游离肺动静脉，可以增加0.9 cm（0.3 cm-1.8 cm）气管切除长度。Rose等^[19]^研究了气管近端的松解方式，包括：气管前后间隙的钝性游离，注意避免伤及气管两侧的血供；舌骨上松解；屈颈，使上部气管进入胸腔，15°-35°可以增加4 cm-5 cm气管切除长度，屈颈 > 35°可以再额外增加1 cm-1.5 cm。

如果吻合口张力仍然很大，其它探索中的减少吻合口张力的方法还有：切除右上肺，将下肺静脉上提吻合重建到上肺静脉回左心房处，这种方式可以增加约1 cm的气管切除长度；切除左全肺，尽量保留长的左主支气管翻转吻合替代气管，相当于50%的气管长度^[20]^；右上叶切除，右主支气管翻转代气管等^[21]^，但由于创伤和手术风险过大，临床上很少用到。

在气管吻合前，术者可以通过上托隆突使气管两断端靠近，直接估计吻合口的张力，一般将1, 000 Gm作为吻合口的张力上限，但在术中直接测量很少用到。手术吻合时，需要在台下协助屈颈，同时由助手向头侧托住右肺和气管隆突，以减少吻合口的张力。

吻合口的情况决定了气管肿瘤切除手术的成败，手术越复杂，术后并发症的发生率越高。在麻省总医院的统计数据^[8]^中，气管切除的并发症发生率为3.9%，而隆突切除的并发症发生率为16%。手术危险因素主要包括：二次手术、糖尿病、切除长度 > 4 cm、第一次切除时行气管造口术^[22]^。其中，第一次手术的成功对患者预后至关重要。

气管环形切除后吻合口张力过大无法直接端端吻合时，就需要有移植物替代气管。Belsey^[23]^最早提出理想的气管移植物的要求：横向有一定硬度，轴向可弯曲性；有纤毛上皮覆盖的表面。Grillo^[24]^总结其后的研究提出，移植物必须完全不透气，以避免局部和纵隔感染；能够与周围组织相容，以避免慢性炎症、肉芽组织生成阻塞管腔、感染和向周围组织侵蚀移位；移植后不需要免疫抑制治疗，因为大部分需要抑制的患者是肿瘤患者；移植手术的过程直接易于操作。

从1979年Rose^[25]^报道第一例气管移植至今，人们一直进行基础和临床的研究，以寻找理想的气管移植物，目前动物试验和临床中应用的气管移植物包括：

人造气管：不锈钢、硅树脂、聚乙烯、聚四氟乙烯树脂、合成树脂等材质的人造气管，有些已用于临床，如Neville人造气管，尽管能在一定时间内保持气道通畅，但最终由于其容易移位、易被肉芽组织阻塞以及与气管吻合处容易感染，限制了其广泛应用，文献还有多例人造气管侵蚀无名动脉致大出血死亡的报道。到现在为止，几乎所有成功的人造移植物，如血管、心脏瓣膜、骨关节等，都是在无菌的部位。在呼吸道、消化道和生殖道尚没有相应成功的人造移植物，原因在于人造物和机体的交界面有慢性修复的结缔组织和上皮细胞，容易被细菌污染，最终感染导致移植失败^[24]^。

尸体气管：Jacob等^[26]^报道24例儿童患者的尸体气管移植术，尸体气管用4%福尔马林和硫柳汞处理并储存于丙酮中，将患者的气管软骨部切除，保留膜部，并移植尸体气管软骨部，管腔内临时置入Bumon或Hood支架，术后需要定期行气管镜清理肉芽组织，术后观察到气管腔内完全再上皮化后取出支架。术后随访5个月-10年，20例患者存活，其中16例没有任何气道问题，4例仍在治疗。然而这些气管更大程度上相当于补片，而不是真正的气管置换，因为它要求保留受体的气管膜部。而且移植物不能随儿童生长而生长，因而远期可能还需要置换。

自体组织：Fonkalsrud对气管发育不全^[27]^和气管狭窄^[28]^的患者用食管代替气管的尝试均不成功。Martinod等^[29, 30]^进行了同体和异体的主动脉移植到气管的动物试验，所有试验羊手术成功，术后病理学检查发现，主动脉发生了转化，首先变成广泛的带有鳞状上皮的炎性组织，术后6个月-36个月逐渐转化成分化良好的气管组织，甚至还包含有粘液纤毛上皮及新形成的软骨环。基于动物试验，Azorin等^[31]^于2006年进行了第一例人气管肿瘤切除、肾下腹主动脉置换手术，切除气管长度7 cm，术中动脉内放置硅酮支架支撑。患者术后早期恢复顺利，术后6个月死于急性肺感染，但是没有吻合口瘘和移植物破裂的表现。Wurtz等^[32]^在《新英格兰医学杂志》报道了2例动脉移植代气管病例，随访1年已发现呼吸道上皮形成。随访1年半，患者没有接受免疫抑制治疗，移植物没有缺血、感染、排斥等反应，但是否有动物试验^[33]^观察到的软骨形成还有待观察。随后动物试验发现，冻存的动脉同新鲜动脉一样，可以转化为气管，而且具有更多的优势：包括可以保存在组织库，永久保存，不需要免疫抑制等。Wurtz等^[34, 35]^报道了6例动脉移植代气管病例，其中前2例使用新鲜动脉，后4例使用冻存动脉，所有移植的动脉都转化为血供丰富的管状组织，并且有呼吸道上皮覆盖，目前后4例患者仍无病生存，且其中3例已正常工作，气管内支架仍未取出，作者希望能有新生软骨生成后足以支撑气管再将支架取出，是否有新生的气管软骨生成还有待随访观察。

组织工程气管：随着组织工程学的发展，在体外通过组织工程学的方法制造出人造气管以供移植成为可能。2008年，西班牙学者Macchiarini等^[36]^在《柳叶刀》杂志上报道了世界上首例组织工程气管移植的病例，患者是一名30岁的女性，患有终末期气管软化症，通过将6 cm长的尸体气管脱细胞和去抗原处理后，把患者的干细胞培养分化的上皮细胞和软骨细胞种于其上，共同培养形成人造气管，然后移植置换患者病变气管，术后患者恢复良好。但整个组织工程气管的生产需要近三个月，无法满足很多急需气管移植的患者。随后，Baiguera等^[37]^改进了去细胞过程，将生产周期缩短到约3周。2010年3月，英国医生更进一步为一名患有长段先天性气管狭窄的10岁儿童直接进行了组织工程气管移植，然后将患者自身的骨髓干细胞直接注射到移植的气管上，让干细胞在体内原位分化成气管软骨成分和上皮成分^[38]^。

对于术后复发的气管肿瘤的再手术治疗的适应症，张逊等^[39]^提出以下几点体会：一是第一次手术时切除气管的长度 < 3 cm；二是复发肿瘤 < 2 cm；三是CT检查提示复发肿瘤与周围组织无明显外侵粘连；四是肿瘤的病理类型为低度恶性者。二次手术可选用经原剖胸切口做气管袖式切除术或气管肿瘤局部切除术。

## 5 原发性气管肿瘤的放疗

对于不宜手术治疗的原发性气管肿瘤，只要患者条件允许，都应进行根治性放疗，一般而言气管腺样囊性癌对放射线比较敏感，鳞癌次之^[6]^。

术前新辅助放疗不被推荐，因为术前放疗影响支气管的血供，使吻合口愈合延迟，并增加导致吻合口裂开风险，特别是对于气管腔外肿物侵犯广泛而需要综合治疗的患者。如果患者进行术前放疗，则需要采取特别措施促进吻合口愈合，包括使用未受放射的血管丰富组织，如带蒂的大网膜包裹吻合口^[40]^。

对于不完全切除的中低分化的恶性肿瘤，推荐术后补充放疗。而高分化的肿瘤，如类癌和粘液表皮样癌，则不推荐放疗。考虑到吻合口的愈合，一般术后2个月以后才开始放疗，放射剂量一般为50 Gy-70 Gy ^[41]^。

## 6 原发性气管肿瘤的其它治疗

气管肿瘤无手术治疗适应症者，为了减轻气道阻塞和肿瘤出血，可行气管镜下YAG激光电灼治疗、冷冻治疗以及气管腔内支架置入等姑息治疗^[6]^。

## 7 总结

综上所述，原发性气管肿瘤是临床上非常少见的肿瘤，由于其临床特征缺乏特异性，常被误诊为肺部感染和支气管哮喘。气管肿瘤有恶性、低度恶性及良性三种，其中良性肿瘤的比例不到10%。气管肿瘤的治疗是以外科手术为主的综合治疗。虽然，目前在气管移植和人造气管研究等方面取得了一定的进展，但在作为临床常规应用上还存在一定的距离。因此，进一步寻找合适的人造材料来代替气管的基础和临床研究必将为气管肿瘤的治疗提供科学依据。
